# A case of superficial pemphigus induced by sodium valproate

**DOI:** 10.1093/omcr/omaf196

**Published:** 2025-10-22

**Authors:** Meryeme Boutaarourt, Ouiame El Jouari, Fatima Zohra El Ali, Salim Gallouj

**Affiliations:** Department of Dermatology-Venereology, Mohamed VI University Hospital, M3MF+GCG, La Nouvelle Ville Ibn Batouta, Tangier 90100, Morocco; Faculty of Medicine and Pharmacy, Abdelmalek Essaadi University, M3MF+GCG, La Nouvelle Ville Ibn Batouta, Tangier 90100, Morocco; Department of Dermatology-Venereology, Mohamed VI University Hospital, M3MF+GCG, La Nouvelle Ville Ibn Batouta, Tangier 90100, Morocco; Faculty of Medicine and Pharmacy, Abdelmalek Essaadi University, M3MF+GCG, La Nouvelle Ville Ibn Batouta, Tangier 90100, Morocco; Department of Dermatology-Venereology, Mohamed VI University Hospital, M3MF+GCG, La Nouvelle Ville Ibn Batouta, Tangier 90100, Morocco; Faculty of Medicine and Pharmacy, Abdelmalek Essaadi University, M3MF+GCG, La Nouvelle Ville Ibn Batouta, Tangier 90100, Morocco; Department of Dermatology-Venereology, Mohamed VI University Hospital, M3MF+GCG, La Nouvelle Ville Ibn Batouta, Tangier 90100, Morocco; Faculty of Medicine and Pharmacy, Abdelmalek Essaadi University, M3MF+GCG, La Nouvelle Ville Ibn Batouta, Tangier 90100, Morocco

**Keywords:** induced superficial pemphigus, iatrogenic pemphigus, sodium valproate, corticosteroid therapy

## Abstract

Iatrogenic pemphigus is a rare entity in routine dermatological practice, particularly in its superficial clinical form. We present a case of superficial pemphigus induced by sodium valproate. Our patient, a 70-year-old female, was being managed for epilepsy with sodium valproate. The interval between the initiation of treatment and the onset of symptoms was approximately one year. Clinical examination revealed oozing erythroderma with blisters and post-bullous erosions predominantly localized to seborrheic areas, along with a positive Nikolsky sign. Histological examination, complemented by direct immunofluorescence, confirmed the diagnosis of superficial pemphigus. Based on clinical findings, histological results, and a review of the literature, the diagnosis of sodium valproate-induced superficial pemphigus was established. The patient achieved remission following the discontinuation of sodium valproate and the initiation of systemic corticosteroid therapy. While cases of drug-induced pemphigus have been documented, this is, to our knowledge, the first report implicating sodium valproate in our department. Identifying the causative drug in drug-induced pemphigus can be challenging, particularly in polymedicated patients, as the latency period between drug initiation and symptom onset can vary widely. Drug-induced pemphigus should be considered in any patient presenting with pemphigus, especially in elderly individuals undergoing multiple treatments. In most cases, withdrawal of the offending drug combined with systemic corticosteroid therapy results in clinical improvement.

## Introduction

Pemphigus is a group of rare autoimmune blistering diseases characterized by intraepidermal blister formation secondary to a loss of adhesion between keratinocytes (acantholysis), mediated by autoantibodies primarily targeting desmogleins. Several clinical forms of pemphigus have been described, among which the so-called ‘superficial’ pemphigus accounts for approximately 20% of all cases [1]. This subgroup mainly includes two clinical entities: seborrheic pemphigus, a localized form that predominantly affects sebaceous-rich areas, and pemphigus foliaceus, the disseminated form that typically involves the trunk, face, and scalp, without mucosal involvement [1, 2].

From an epidemiological perspective, pemphigus affects both sexes, with a female predominance observed in some series and a peak incidence around the fifth decade of life [2]. Although superficial forms are considered less severe than pemphigus vulgaris, they can cause significant functional and aesthetic impairment, thus requiring prompt and adequate management.

A particular variant of the disease is drug-induced pemphigus, also known as iatrogenic pemphigus. Since its first description in the 1950s, approximately 200 cases have been reported in the medical literature [3]. The most frequently implicated agents include thiol-containing drugs, such as penicillamine and captopril. However, non-thiol drugs, including certain antibiotics, anti-inflammatory agents, angiotensin-converting enzyme inhibitors, and anticonvulsants, have also been associated [3, 4]. Sodium valproate, a widely prescribed antiepileptic drug, is only rarely associated with autoimmune dermatologic conditions, and its implication in pemphigus remains exceptionally reported.

The pathophysiological mechanisms underlying drug-induced pemphigus are not fully understood. Several hypotheses have been proposed, including the possibility that drugs or their metabolites act as haptens modifying desmosomal antigens, induce molecular mimicry, or disrupt immune tolerance, thereby promoting an autoimmune response against desmogleins. Genetic and environmental predispositions may also contribute to these aberrant immunological responses [4, 5].

The diagnosis of superficial pemphigus relies on a combination of clinical, histopathological, and immunological findings. Clinically, it manifests as superficial, flaccid blisters that rupture rapidly, leaving crusted erosions, often beginning in seborrheic areas before becoming more widespread. Histology typically reveals a subcorneal intraepidermal blister with acantholysis. Direct immunofluorescence of perilesional skin demonstrates intercellular deposits of IgG and/or C3. Indirect immunofluorescence often reveals circulating autoantibodies directed against desmoglein 1, the main antigen involved in superficial forms [2, 6].

Management of drug-induced pemphigus primarily involves prompt identification and discontinuation of the offending drug, which may lead to spontaneous improvement in some cases. Nonetheless, systemic therapy with corticosteroids and/or immunosuppressive agents is often required, especially in severe or persistent cases [3].

Herein, we report a case of superficial pemphigus induced by sodium valproate in a patient without any significant dermatological history, who had been on long-term treatment for a neurological disorder. This case is noteworthy for two main reasons: first, due to the rarity of sodium valproate being implicated in the etiology of pemphigus, and second, because of the clinical presentation consistent with a disseminated superficial form, confirmed by immunopathological investigations. This report aims to expand current knowledge on drug-induced pemphigus and to highlight the importance of thorough medication history in the evaluation of any newly emergent bullous dermatosis.

## Case report

**Figure 1:**
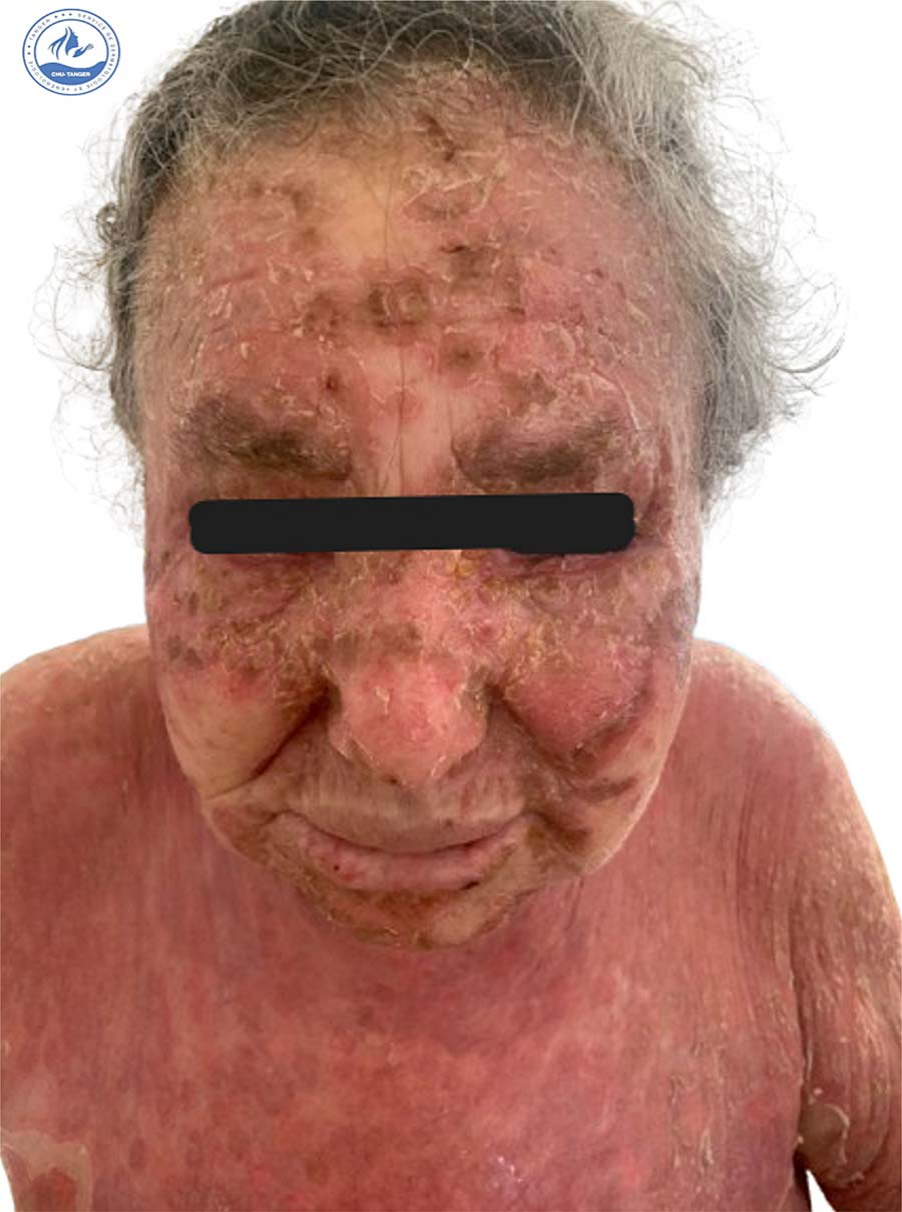
Clinical presentation at admission showing erythematous-squamous plaques covered with meliceric crusts on the face.

**Figure 2:**
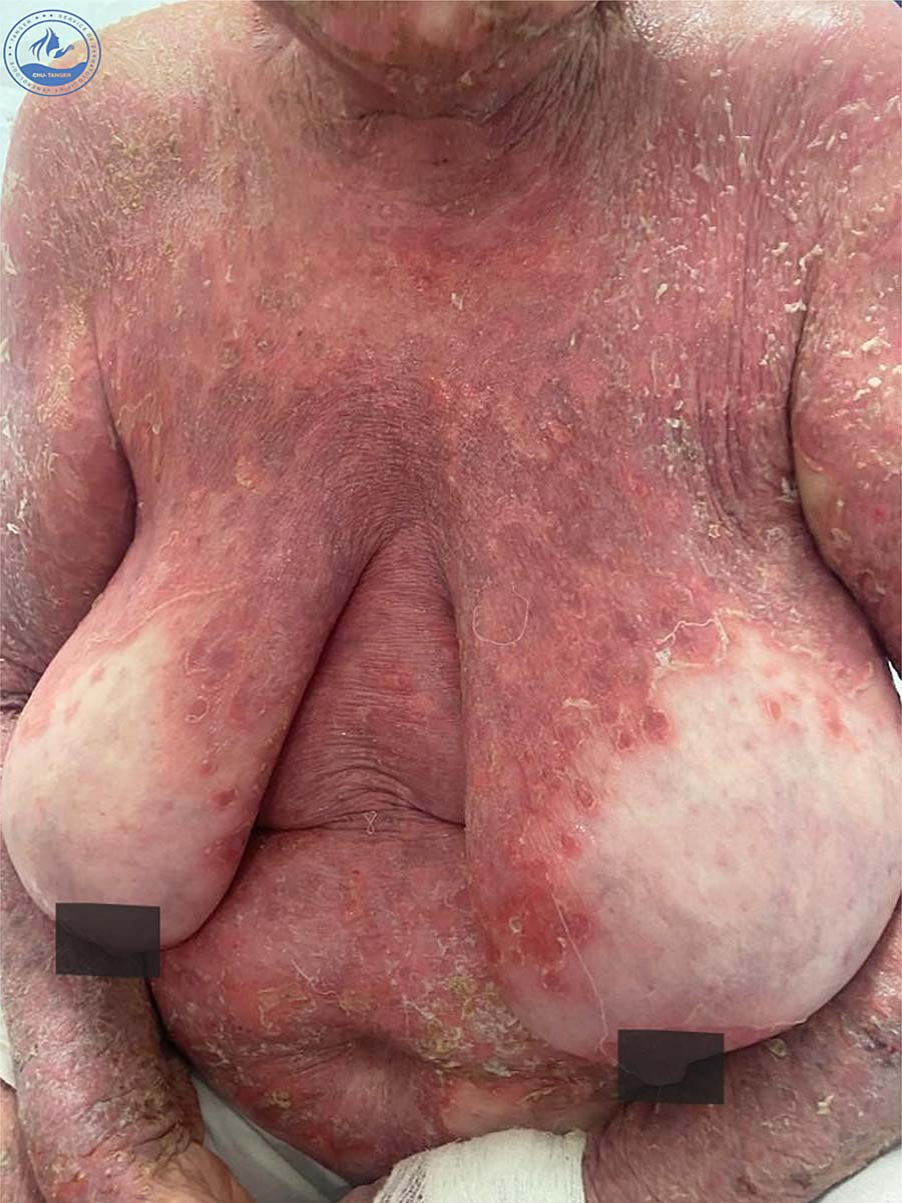
Clinical presentation at admission showing erythematous-squamous plaques covered with meliceric crusts on the trunk.

**Figure 3:**
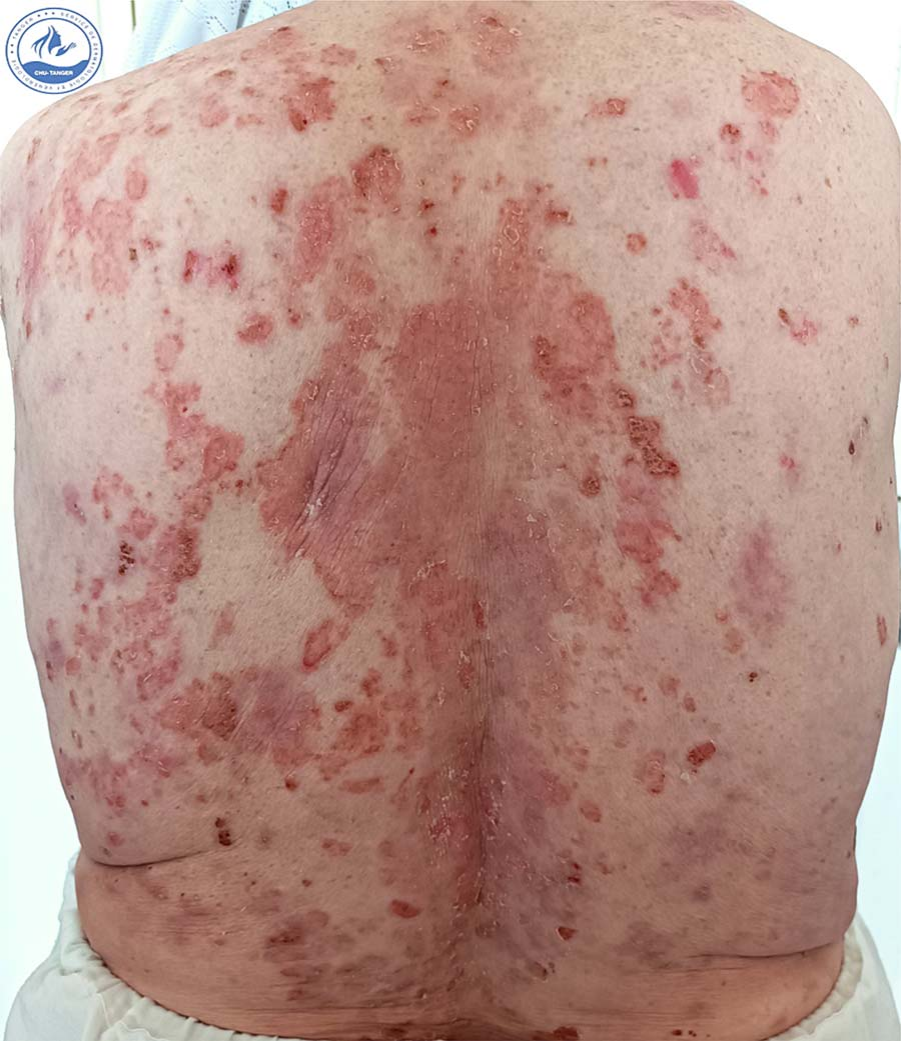
Clinical presentation at admission showing erythematous plaques and post-bullous erosions exposing a pink surface bordered by an epithelial collar on the back.

We present a 70-year-old female patient with a history of epilepsy, treated with sodium valproate 500 mg/day for one year, who was admitted for the management of an oozing erythroderma that had evolved over the past few weeks, associated with fever and general malaise. Upon dermatological examination at admission, the patient presented with erythematous, confluent plaques and post-bullous erosions, covering approximately 92% of the skin surface. The lesions were more pronounced on the face (Fig. 1) and trunk (Fig. 2). Some areas were covered with hemorrhagic and meliceric crusts, while others exposed a pink surface bordered by an epithelial collar (Fig. 3). The oral, ocular, and anogenital mucous membranes were spared. Three 1 cm bullae with clear fluid were observed on the right knee, and a positive Nikolsky sign was present.

**Figure 4:**
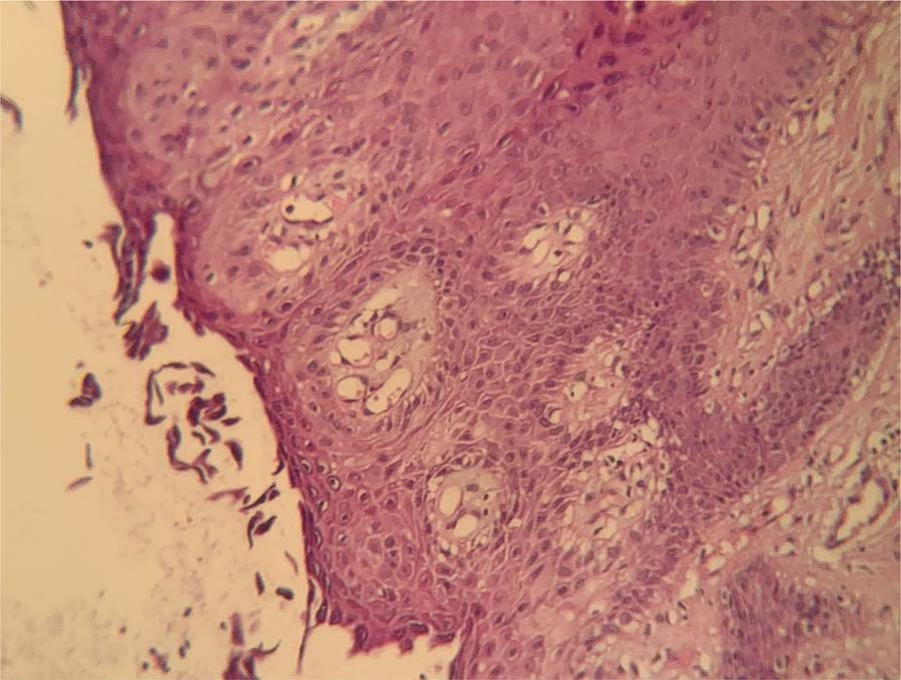
Histological aspect revealing a superficial subcorneal epidermal cleavage consistent with superficial pemphigus.

**Figure 5:**
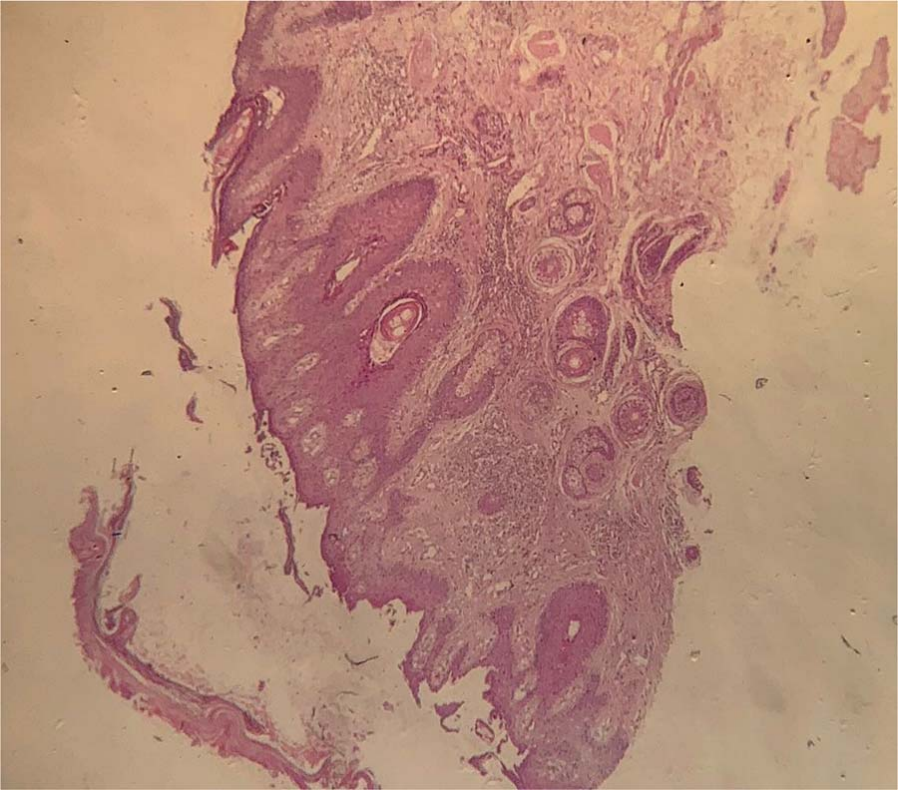
Histological aspect showing extensive subcorneal epidermal detachment with absence of a complete blister (H&E stain x10).

**Figure 6:**
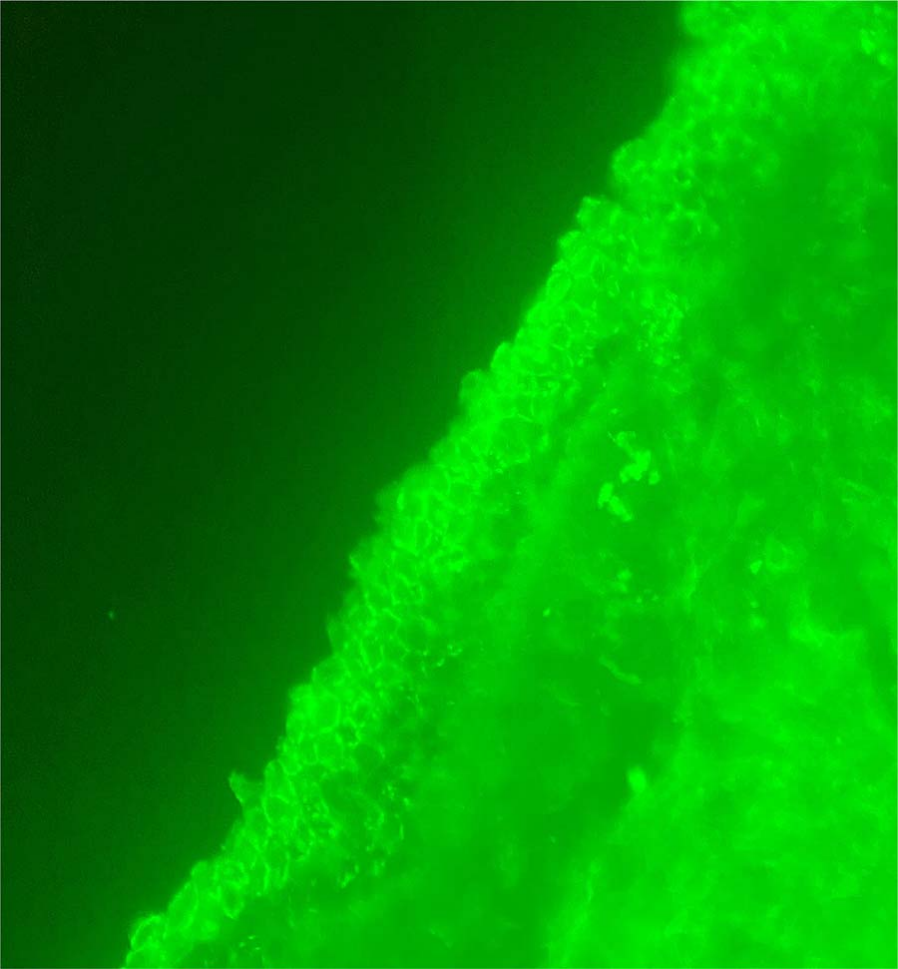
Direct immunofluorescence revealing intercellular deposits of IgG and C3 in a net-like pattern, predominantly in the superficial layers of the epidermis.

Histological examination (Figs 4, 5) of the skin biopsy revealed a subcorneal intraepidermal cleft, associated with scattered acantholytic keratinocytes. The epidermis appeared eroded in some areas, with residual keratinocyte debris and focal hyperkeratosis. Moderate spongiosis was present. The superficial dermis showed a mild perivascular inflammatory infiltrate, composed mainly of lymphocytes with a few eosinophils. Direct immunofluorescence (Fig. 6) performed on perilesional skin demonstrated intercellular deposits of IgG and C3 in a net-like pattern, predominantly in the superficial layers of the epidermis. Indirect immunofluorescence targeting intercellular substance was positive, as were anti-desmoglein 1 antibodies, while anti-desmoglein 3 antibodies were negative. These findings were consistent with superficial pemphigus.

After consultation with neurology, the offending drug was replaced with levetiracetam 500 mg/day, and the case was reported to the pharmacovigilance center. The patient was treated with oral corticosteroids (1 mg/kg/day) following a 3-day intravenous bolus of 1 g/day.

**Figure 7:**
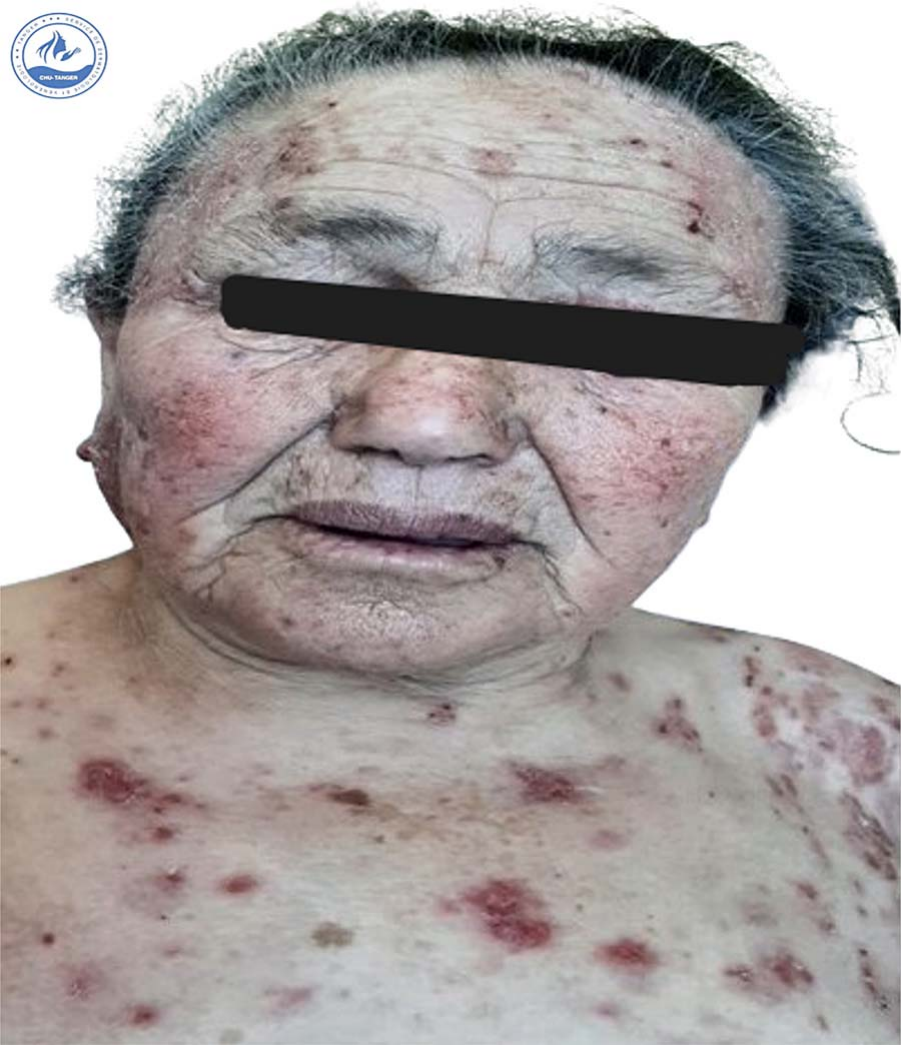
Clinical aspect one week after discontinuation of the implicated drug.

**Figure 8:**
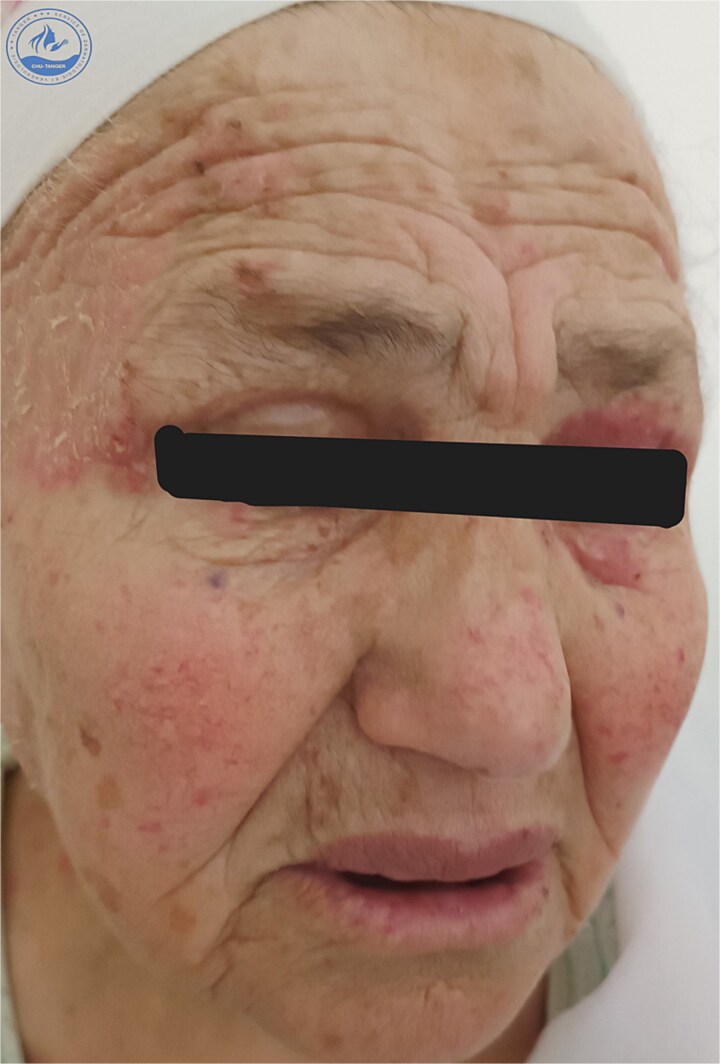
Clinical aspect three weeks after discontinuation of the implicated drug.

The clinical course showed significant improvement (Figs 7, 8) within weeks after discontinuation of the suspected medication and initiation of corticosteroid therapy.

A thorough review of the case in collaboration with the pharmacovigilance center confirmed the causality of sodium valproate in the development of superficial pemphigus in this patient. The temporal relationship between the initiation of the medication and the onset of symptoms (1 year) was consistent with similar cases reported in the literature. Rapid remission following drug discontinuation and corticosteroid treatment further supported the diagnosis of drug-induced pemphigus rather than spontaneous pemphigus. The Naranjo scale, which considers factors such as drug timing, reversibility upon discontinuation, and other contributing factors.

## Discussion

Pemphigus is an intraepidermal and/or intraepithelial autoimmune blistering disease characterized by a loss of adhesion between keratinocytes. This is mediated by autoantibodies targeting desmoglein 1 (Dsg1) in superficial pemphigus or desmoglein 3 (Dsg3) in pemphigus vulgaris, both of which are transmembrane proteins integral to desmosomes [1]. In its superficial form, pemphigus includes seborrheic pemphigus and pemphigus foliaceus (as observed in our patient), which are characterized by exclusive skin involvement, subcorneal cleavage, and autoantibodies directed against desmoglein 1 [1]. The clinical presentation typically includes erythematous-squamous plaques resulting from transient superficial blisters. Pruritus is often described, along with a positive Nikolsky sign. Mucosal surfaces are generally spared. The transient blisters are quickly replaced by squamous or crusted plaques, which may coalesce, leading to oozing erythroderma.

Drug-induced pemphigus, while rare, is an important entity to consider, particularly in patients undergoing chronic treatment. The implication of sodium valproate as an inducer, as in this case, is notable. Sodium valproate, a widely used anticonvulsant, is known for its diverse side effects, but its association with blistering diseases is exceptional and, to the best of our knowledge, not previously documented in the literature, highlighting the uniqueness of this case. Drugs implicated in 80% of drug-induced pemphigus cases typically contain thiol groups, such as captopril [7]. Other drugs, including beta-lactams, nevirapine, oxicam, phenobarbital, and propranolol, have also been reported as triggers.

The underlying mechanisms of drug-induced pemphigus are multifactorial and remain a subject of debate. Proposed mechanisms include; Molecular mimicry: the drug or its metabolite may induce an autoimmune response by mimicking desmosomal antigens (notably desmoglein 1 or 3), leading to the production of autoantibodies [8]. Antigenic modification: the drug may alter the structure of desmosomal antigens, rendering them immunogenic to the immune system [8]. Non-specific immune activation: certain drugs, such as anticonvulsants, may disrupt immune homeostasis, provoking autoantibody production [8].

The clinical presentation of drug-induced pemphigus includes flaccid blisters or post-bullous erosions on erythematous skin, often with a positive Nikolsky sign. Mucosal involvement is more typical in this form of pemphigus [9]. Diagnosis can be challenging due to the variability in latency periods between drug exposure and symptom onset, which may range from weeks to years. In this case, the one-year interval aligns with reported delays for other drugs implicated in pemphigus [7].

Diagnostic confirmation relies on correlating clinical findings with histology and direct immunofluorescence, which demonstrate immunoglobulin G (IgG) or C3 deposits in keratinocytes. However, establishing drug causality can be difficult, particularly in polymedicated patients. Tools such as the Naranjo Adverse Drug Reaction Probability Scale may aid in strengthening causal hypotheses [9].

Management involves discontinuing the implicated drug and initiating systemic corticosteroid therapy. In this case, remission was rapidly achieved following the cessation of sodium valproate, underscoring the critical role of drug withdrawal in clinical improvement [9]. Nonetheless, spontaneous regression after drug discontinuation is not always observed and may necessitate prolonged corticosteroid therapy to prevent relapses, especially in cases where circulating autoantibodies persist. Immunosuppressive agents such as azathioprine, methotrexate, or cyclophosphamide are also used in severe forms of the disease. The use of an anti-CD20 antibody, rituximab, has also proven to be a promising therapy for refractory pemphigus in a number of cases, by targeting aberrant B cells [9]. Overall, there is a lack of well-controlled clinical studies on the appropriate treatment of drug-induced pemphigus, but the cornerstone of therapy remains corticosteroid treatment.

Although over 200 cases of drug-induced pemphigus have been reported, particularly with angiotensin-converting enzyme inhibitors [7], the association with sodium valproate has not been previously described, adding significant value to the medical literature. Sodium valproate’s mechanism of action, involving ion channel modulation and neurotransmitter regulation, may indirectly contribute to the observed immune activation. This case enriches existing data by expanding the list of potential triggers and emphasizes the importance of a thorough medication history in any patient presenting with pemphigus, particularly in elderly patients on chronic therapy. Early recognition of drug-induced pemphigus can prevent unnecessary escalation of therapy and improve prognosis by promptly discontinuing the offending agent.

## Conclusion

This case highlights a novel association between sodium valproate and superficial pemphigus, contributing to the expanding knowledge of drug-induced pemphigus. To the best of our knowledge, this is the first published report linking sodium valproate to superficial pemphigus. It also underscores the importance of interdisciplinary collaboration in the diagnosis and management of such rare conditions.

Clinicians should maintain a high level of vigilance, as identifying a drug-related etiology can transform management and prevent complications associated with prolonged treatment. The management of drug-induced pemphigus primarily involves discontinuing the implicated drug, initiating systemic corticosteroid therapy, and reporting the case to pharmacovigilance centers.
